# *groEL* Gene-Based Phylogenetic Analysis of *Lactobacillus* Species by High-Throughput Sequencing

**DOI:** 10.3390/genes10070530

**Published:** 2019-07-12

**Authors:** Miaoqi Xie, Mingluo Pan, Yang Jiang, Xiaoming Liu, Wenwei Lu, Jianxin Zhao, Hao Zhang, Wei Chen

**Affiliations:** 1 State Key Laboratory of Food Science and Technology, Jiangnan University, Wuxi 214122, China; 2 School of Food Science and Technology, Jiangnan University, Wuxi 214122, China; 3 National Engineering Research Center for Functional Food, Jiangnan University, Wuxi 214122, China; 4 Yangzhou Institute of Food Biotechnology, Jiangnan University, Yangzhou 225004, China; 5 Wuxi Translational Medicine Research Center and Jiangsu Translational Medicine Research Institute Wuxi Branch, Wuxi 214122, China; 6 Beijing Innovation Centre of Food Nutrition and Human Health, Beijing Technology and Business University (BTBU), Beijing 100048, China

**Keywords:** *Lactobacillus*, *groEL*, phylogenetic analysis, species diversity, MiSeq Illumina sequencing platform

## Abstract

*Lactobacillus* is a fairly diverse genus of bacteria with more than 260 species and subspecies. Many profiling methods have been developed to carry out phylogenetic analysis of this complex and diverse genus, but limitations remain since there is still a lack of comprehensive and accurate analytical method to profile this genus at species level. To overcome these limitations, a *Lactobacillus*-specific primer set was developed targeting a hypervariable region in the *groEL* gene—a single-copy gene that has undergone rapid mutation and evolution. The results showed that this methodology could accurately perform taxonomic identification of *Lactobacillus* down to the species level. Its detection limit was as low as 10^4^ colony-forming units (cfu)/mL for *Lactobacillus* species. The assessment of detection specificity using the *Lactobacillus* *groEL* profiling method found that *Lactobacillus, Pediococcus, Weissella,* and *Leuconostoc* genus could be distinguished, but non-*Lactobacillus* Genus Complex could not be detected. The *groEL* gene sequencing and Miseq high-throughput approach were adopted to estimate the richness and diversity of *Lactobacillus* species in different ecosystems. The method was tested using kurut (fermented yak milk) samples and fecal samples of human, rat, and mouse. The results indicated that *Lactobacillus mucosae* was the predominant gut *Lactobacillus* species among Chinese, and *L. johnsonii* accounted for the majority of lactobacilli in rat and mouse gut. Meanwhile, *L. delbrueckii* subsp. *bulgaricus* had the highest relative abundance of *Lactobacillus* in kurut. Thus, this *groEL* gene profiling method is expected to promote the application of *Lactobacillus* for industrial production and therapeutic purpose.

## 1. Introduction

*Lactobacillus* genus is a Gram-positive, non-spore-forming, catalase-negative, rhabditiform, facultative anaerobic or microaerophilic group of bacteria [1]. According to the fermentation products, *Lactobacillus* species can be segmented into homofermentative and heterofermentative lactobacilli. Due to fastidious nutritional requirement, *Lactobacillus* lives in nutrient-rich environment, such as fermented or spoiled foods, animal feed, plant and soil surfaces, and bodies of invertebrate and vertebrate animals [2]. *Lactobacillus* species are also indigenous to human digestive, urinary, and genital systems [3]. Although they usually protect human from pathogen invasion, they can be occasional opportunistic pathogens [4]. Some *Lactobacillus* strains are probiotic and serve as supplements for the prevention and treatment of various gastrointestinal diseases [5]. Thus, assessment of the diversity of *Lactobacillus* species in different ecological environment is expected to provide valuable reference to promote the application of the bacteria for industrial production and therapeutic purpose.

In taxonomic perspective, *Lactobacillus* is an extremely diverse genus comprising of 240 species and 29 subspecies (http://www.bacterio.net/lactobacillus.html), greatly exceeding the diversity of a typical bacterial family [6]. However, with the unreliability of biochemical identification methods, the molecular biology methods have been proved to be effective for differentiating *Lactobacillus* species [7]. For instance, Zheng et al. used the core and pan-genomes of 174 *Lactobacillus and Pediococcus* strains and separated these strains into 24 phylogenetic groups [8]. A cladogram of 452 genera [9] showed that the *Lactobacillus* clade includes species from six different genera (*Lactobacillus, Pediococcus, Weissella, Leuconostoc, Oenococcus,* and *Fructobacillus*), and it was proposed to name these six genera as constituting the *Lactobacillus* Genus Complex. 16S rRNA profiling methods have been widely used to assess microbial flora but it is also known that several closely related species cannot be correctly differentiated. A profiling method based on an internal transcribed spacer (ITS) has been proposed to identify *Lactobacillus* down to species level, but limitations remain because only 62 species had complete *Lactobacillus* ITS amplicon (LITSA) sequences, among which 18 species had an average LITSA sequence identity of above 97% compared with one other *Lactobacillus* species [10]. Phylogenetic inference can also be made from several genes or proteins, such as *groEL*, *rplB*, *rpoB*, *recA*, *tuf*, *aroE*, *ddl*, *dnaE*, *fusA*, *ftsZ*, *glnA*, *gyrB*, *glpF*, *gltX*, *gyrB*, *gpd*, *gdh*, *hemN*, *ileS*, *lepA*, *leuS*, *ldhL*, *mutS*, *mutL*, *metRS*, *nrdD*, *pepV*, *pgm*, *polA*, *recG*, *recP*, *xpt*, *yqil*, *tkt*, and *tpi* [7,11,12]. Some of these genetic markers have shown the ability to identify *Lactobacillus* species with the same or even better accuracy and sensitivity than 16S rRNA gene [11]. Among these molecular markers, *groEL* gene, which translates the synthesis of heat stress proteins was used to identify lactic acid bacteria as early as ten years ago [13,14]. Reportedly, *groEL* gene can achieve far better resolution than 16S rRNA gene, and topological structure of the phylogenetic tree using this gene is the same as that for the 16S rRNA gene [11]. Meanwhile, *groEL* gene has a high rate of evolution [15] and is single copy, commonly found in lactobacilli [16], and many lactobacilli gene sequences are available in the chaperonin sequence database (cpnDB). This facilitates the identification and quantitative analyses of this genus. In the meantime, the molecular biological identification methods when combined with the error-proof, high throughput next-generation sequencing technologies may facilitate phylogenetic analysis of microbiota, which had previously been used to analyze the *Bifidobacterium* genus [15,17].

This study aimed to introduce a novel profiling protocol to assess *Lactobacillus* species using the distinguishing marker *groEL* gene and the MiSeq Illumina sequencing platform. A new primer set was also designed and its robustness tested in identifying *Lactobacillus* species in mammalian fecal and kurut (fermented yak milk) samples.

## 2. Materials and Methods

### 2.1. Bacterial Culture and Genome Extraction

A list of 35 *Lactobacillus* strains employed in this research is shown in Table S1 in the Supplementary Materials. The growth conditions of all *Lactobacillus* strains were based on previous literature report [18]. The following 12 non-*Lactobacillus* strains were also used: *Streptococcus thermophiles* ST2017, *Escherichia coli* BL21, *Bacteroides ovatus* ATCC8483, *Bifidobacterium longum* CCFM861, *Bi. animalis* CCFM624, *Bi. breve* CCFM684, *Bi. adolescentis* CCFM626, *Leuconostoc mesenteroides* NT5-1, *Leuconostoc lactis* DYNDL 7-2, *Pediococcus acidilactici* HN17-2, *Pediococcus pentosaceus* H27-1L, and *Weissella cibaria* FSDLZ2M11. *S. thermophilus* was grown in lactic acid medium at 42 °C; *E. coli*, *Ba. ovatus,* and all *Bifidobacterium* strains were cultured according to the literature [15,19,20]. *Leuconostoc mesenteroides*, *Leuconostoc lactis*, *Pediococcus acidilactici*, *Pediococcus pentosaceus*, and *Weissella cibaria* were grown in de Man–Rogosa–Sharpe (MRS) medium with 0.05% (wt/vol) L-cysteine hydrochloride in an anaerobic environment at 37 °C. All bacterial strains employed in the research were obtained from the Culture Collection of Food Microorganisms of Jiangnan University (Wuxi, China).

The genomes of all above bacterial strains were extracted and purified as described earlier [21].

### 2.2. Sample Collection and Genome Extraction

In this study, six human, six rat, and six mouse fecal samples were collected. The methods of collection, transportation, and storage of the stool samples were reported previously [15]. The bacterial genome DNA of these samples were extracted with FastDNA SPIN Kit for Feces (MP Biomedicals, Carlsbad, CA, USA) in accordance with producer’s suggestions.

Traditional fermented yak milk samples were retrieved from the herdsmen’s homes in the pasture region of Qinghai, China. The samples were collected, transported, and stored as reported previously [22]. The genomic DNA from these kurut samples was extracted using FastDNA Spin Kit for Soil (MP Biomedicals, Carlsbad, CA, USA) in accordance with producer’s instruction.

All procedures followed in this research were authorized by the Ethical Committee of Jiangnan University (Wuxi, China).

### 2.3. Selection of Lactobacillus groEL Gene

The sequences of 73 *Lactobacillus* core genes based on a previous study [9] were retrieved from the National Center for Biotechnology Information (NCBI) genome database and European Molecular Biology Laboratory (EMBL) database. With MEGA7.0 software, the sequences of genome were aligned using ClustalW program, and phylogenetic trees were established using maximum likelihood (ML) method with a supporting bootstrap value of 1000. The resulting gene sequences were filtered based on the results in the literature [15,23]. Accordingly, *groEL* gene was chosen as the marker gene for sequencing analysis of the *Lactobacillus* strains.

### 2.4. Phylogenetic Analysis

Phylogenetic trees on the basis of *groEL* gene and the V3–V4 region of 16S rRNA gene of *Lactobacillus* strains (Table S4) were constructed using MEGA7.0 software [24,25]. The ClustalW program was used for sequence alignment [26], and maximum likelihood (ML) method was used to yield phylogenetic trees with bootstrap support value of 1000.

### 2.5. Lactobacillus groEL-Specific Primer Design

The available *groEL* gene sequences of 108 species of *Lactobacillus* (Table S2) were downloaded from the NCBI and EMBL databases, and sequences alignment was conducted using the ClustalW program [26]. The results of the analysis were then used to guide the designing of primers using the PRIMER [27] and OLIGO [28] software. The sequences of designed degenerate primers were as follows: forward primer Lac_*groEL*_F, 5′-GCYGGTGCWAACCCNGTTGG-3′ and reverse primer Lac_*groEL*_R, 5′-AANGTNCCVCGVATCTTGTT-3′. The WebLogo pictures of primers were made using the website (http://weblogo.berkeley.edu/) [10].

Shanghai Shengong Biological Engineering Co., Ltd. (Shanghai, China) was responsible for the synthesis of the designed primers.

### 2.6. Evaluation of the Accuracy and Specificity of the Degenerate Primers

A mock *Lactobacillus* community was prepared using nine *Lactobacillus* strains (Table S3) that were cultured separately on MRS medium in an anaerobic atmosphere until they reached the late log phase. The DNA extraction methods of these strains were consistent with those reported in previous literature [15] and mixed in a ratio of 0.01 ng to 50 ng. The colony-forming units (cfu) of the *Lactobacillus* strains were calculated based on the *groEL* gene copy numbers by the means of a calculator provided by the URI Genomics & Sequencing Center (http://cels.uri.edu/gsc/cndna.html). Subsequently, PRIMER-BLAST was applied to perform specific testing by in silico PCR amplification, and NCBI non-redundant (nr) database was used as a reference database [29]. Three parallel experiments were carried out.

### 2.7. PCR Conditions, Quantifiable and Sequencing Measures

The extracted DNA of 35 *Lactobacillus* strains and 12 non-*Lactobacillus* strains described above were used for PCR amplification. The PCR conditions used in *groEL* profiling were as followed: 5 min at 95 °C, followed by 35 cycles of 30 s at 95 °C, 30 s at 58 °C, 60 s at 72 °C, and final elongation at 72 °C for 7 min. Furthermore, the DNA extracted from fecal and kurut samples were used for amplification of partial *groEL* gene using the primers Lac_*groEL*_F/Lac_*groEL*_R, as well as the V3–V4 region within 16S rRNA gene and primer pair 341F/806R was used on the basis of previously reported protocol [30]. Further purification and quantification of all PCR amplified products were conducted according to reported protocols [15]. The purified DNA was sequenced by 2 × 300-bp paired-end Illumina sequencing. The DNA amplicons library was made with TruSeq DNA LT Sample Preparation Kit (Illumina, San Diego, CA, USA) and sequenced by MiSeq Illumina platform with the MiSeq v3 Reagent Kit (600 cycles) according to manufacturer’s instructions.

### 2.8. Cross-Alignment Analysis

All available universal target (UT) sequences of *groEL* gene of *Lactobacillus* were retrieved from the chaperonin database, which contains 533 strains correspond to 129 *Lactobacillus* species (Table S5). With MatGAT software, cross-alignment of all sequences was conducted.

## 3. Results

### 3.1. Selection of the Lactobacillus groEL Gene

The sequences of 73 core genes based on previous studies were retrieved for the selection of marker gene for *Lactobacillus* genus. The results showed the following distinct types of core genes: first, core genes without highly variable region sequences and low resolution that could not be used to distinguish between *Lactobacillus* species; second, core genes with highly variable regions and high resolution that could distinguish between different *Lactobacillus* species but their ends did not contain relatively conserved sequences, which restrict the designing of primers; third, core genes with highly variable regions, high resolution to distinguish between *Lactobacillus* species, and relatively conserved sequences at their ends, e.g., the *groEL* gene, thus suitable for designing primers. Therefore, the *groEL* and 16S rRNA gene sequences of all *Lactobacillus* species that deposited in the EMBL and NCBI databases were downloaded and pairwise sequence similarity using BLAST were performed. The results revealed that the lowest and average pairwise similarity values of the *groEL* gene were 69.63% (*L. iners* and *L. ingluviei*) and 79.61%, respectively, whereas those of the 16S rRNA gene were 83.77% (*L. thailandensis* and *L. iners*) and 89.58% respectively. These indicate that the *groEL* gene evolves at a high rate that allows identification of *Lactobacillus* species more accurately when comparing to 16S rRNA. What’s more, discrimination of the 129 species of *Lactobacillus* was achieved based on the cross-alignment of *groEL* sequences (Table S5). As a result, the *groEL* gene was selected as the core gene for identification of *Lactobacillus* species.

### 3.2. Phylogenetic Analysis of the Lactobacillus groEL Gene

**Figure 1:**
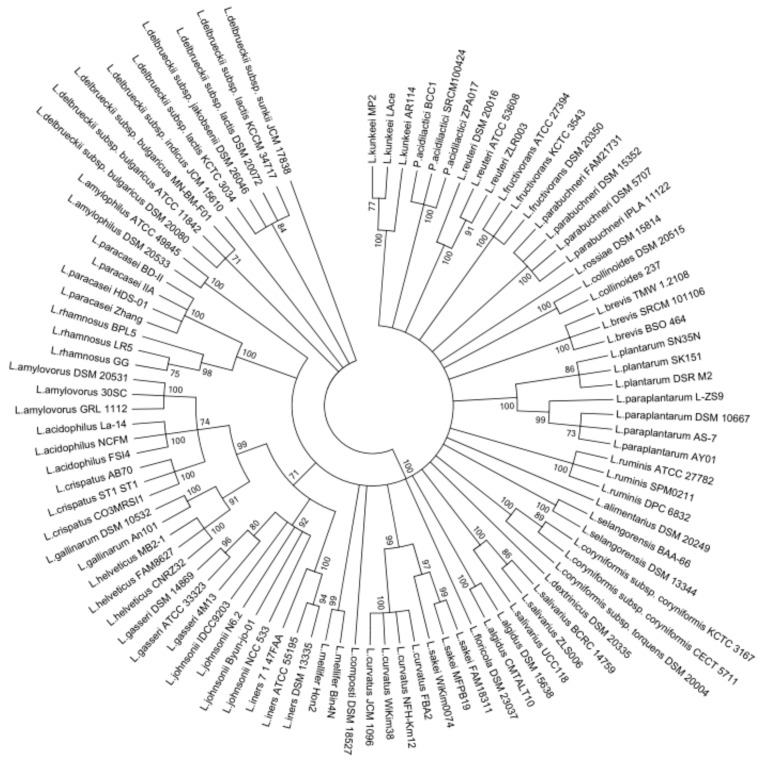
Phylogenetic tree of *Lactobacillus* on the basis of *groEL* gene sequences by maximum likelihood (ML) analysis method. Bootstrapping with 1000 replicates, and bootstrap values over 70% were shown on the branches.

The members of *Lactobacillus* genus were previously separated into 24 phylogenetic groups including *Pediococcus* genus based on the concatenated protein sequences of single-copy core genes. In view of this, a phylogenetic tree was constructed based on the *groEL* gene sequences containing 97 *Lactobacillus* strains and *Pediococcus* strains of 22 phylogenetic groups, using the Maximum Likelihood method with 1000 bootstrap values (Figure 1). According to the phylogenetic tree, *Lactobacillus amylovorus, Lactobacillus acidophilus, Lactobacillus cripatus, Lactobacillus gallinarum, Lactobacillus helveticus, Lactobacillus gasseri, Lactobacillus johnsonii,* and *Lactobacillus iners* were gathered into a large cluster, whereas *Lactobacillus delbrueckii* subspecies were individually assembled into a cluster. Meanwhile, *Lactobacillus ruminis* was separated from the *Lactobacillus salivarius* group when the phylogenetic tree was built on the basis of *groEL* gene. On the other hand, the closely related *Lactobacillus* species and subspecies formed clusters, and distinct *Lactobacillus* species formed different clusters. The *Pediococcus* species were gathered into an individual branch on the phylogenetic tree. However, the phylogenetic dendrogram using the 16S rRNA gene indicated some distinct *Lactobacillus* species were grouped into the same clusters, such as *Lactobacillus iners* and *Lactobacillus amylophilus*, *Lactobacillus brevis* and *Lactobacillus amylophilus* (Figure S1). These results suggest that *groEL* gene could better differentiate between the *Lactobacillus* species when compared to 16S rRNA gene.

### 3.3. Design of Novel Primer Sets for Identifying Lactobacillus

**Figure 2:**
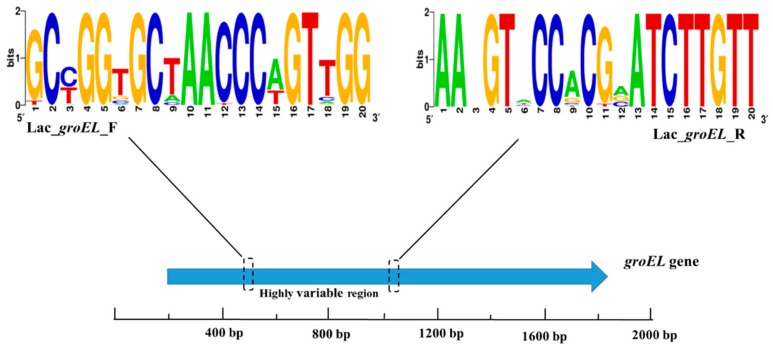
A WebLogo representation was generated to demonstrate the primer sequence conservation. The higher the total stack height, the better the sequence conservativeness of the location. Different symbols at the same location indicate different relative frequencies of the nucleic acid.

The NCBI database and the chaperonin sequence database contain enough *groEL* gene sequences of *Lactobacillus* strains to meet the requirements of sequence alignment and new species identification. The emergence of next-generation sequencing technology facilitates amplification and sequencing of partial gene sequences of bacterium. The full-length *groEL* gene is approximately 1600 bp long, which is not suitable for short-read sequencing platform. Therefore, partial *Lactobacillus groEL* gene was used for sequencing and designing a novel primer set (Lac_*groEL*_F and Lac_*groEL*_R) as per the primer design criteria using the MiSeq Illumina sequencing platform. Based on the results of multiple sequence alignment, the 340–824 bp region of the *groEL* gene was targeted for PCR amplification. After the procedure of PCR, the amplicon was about 480 bp which could be sequenced by 2 × 300-bp paired-end Illumina sequencing. Figure 2 shows the sequence conservation of the *groEL* gene as represented by WebLogo images.

### 3.4. Development of Biological Information Analysis Tool for Lactobacillus

All of the available complete sequences of the *Lactobacillus groEL* gene were downloaded from the NCBI, EMBL, and chaperonin sequence database and summarized in an annotation library (Lac_*groEL*_database). This database is constantly updated with newly obtained sequences of the *Lactobacillus groEL* gene.

Disassembly data were analyzed with the QIIME1.9.1 software. Briefly, disassembly sequences were first screened to select sequences with quality Q value higher than 30, and high-quality sequences were spliced with Flash software package. The splicing condition was overlap base number of >10 bp, and no mismatch base was found. The barcode and primer sequences were then removed to obtain the final effective sequence. Notably, sequence similarity of 97% could be clustered into one operational taxonomic unit (OTU). OTUs based on the gene composition of *Lactobacillus groEL* were comparable with those in Lac_*groEL*_database.

### 3.5. Performance Evaluation of the Degenerate Primers for Identifying Lactobacillus Species

In silico PCR was performed using PRIMER-BLAST for evaluating the specificity of the novel primer set. The result showed that only one amplicon was generated from the *Lactobacillus* genomes, indicating that the primer set had good specificity for *Lactobacillus*. Further, an in vitro experiment was carried out by using the primers Lac_*groEL*_F/Lac_*groEL*_R for amplifying DNA obtained from 35 *Lactobacillus* and 12 non-*Lactobacillus* species. The results showed that PCR product could be obtained using the template DNA extracted from *Lactobacillus*, *Pediococcus*, *Weissella*, and *Leuconostoc* species (Figure S2), indicating that the newly designed primer set could distinguish *Lactobacillus* and genera historically associated with or grouped within the lactobacilli. Other bacteria cannot be amplified by corresponding gene segments in the research.

**Figure 3:**
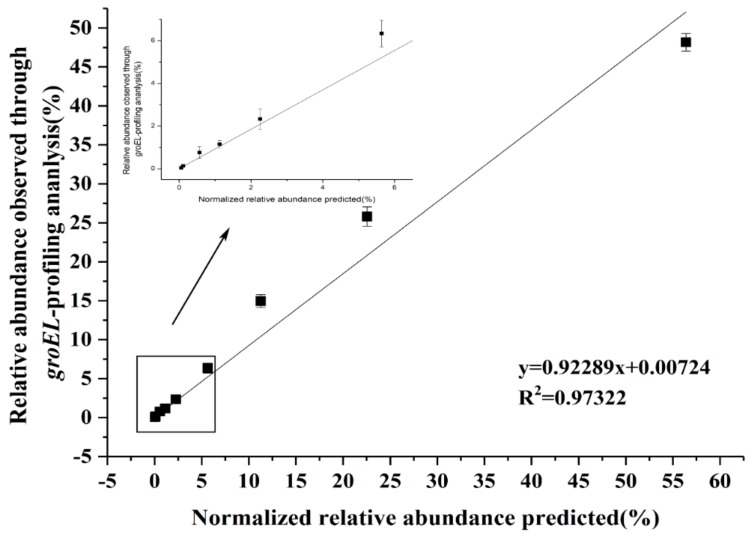
Accuracy and sensitivity of the new primer pair. The relative abundance of the predicted artificial *Lactobacillus* community was normalized, which was well consistent with the results of the analysis method based on the new primers. The linear regression equation is y = 0.92289x + 0.00724, and the y value represents the relative abundance observed through *groEL*-profiling analysis. The correlation index R^2^ = 0.97322.

In addition, the ability to distinguish *Lactobacillus* accurately and sensitively by the new degenerate primers were evaluated. Briefly, the DNA of different *Lactobacillus* species were mixed in known quantities. After PCR amplification with new primers for this artificial *Lactobacillus* community, the amplicons were sequenced using MiSeq Illumina sequencing platform. The findings indicated that the predicted relative concentration of *Lactobacillus* had a good correlation with the relative concentration measured using the degenerate primer pair Lac_*groEL*_F/Lac_*groEL*_R, proving the accuracy of the new primers (Figure 3). The lowest amount of *Lactobacillus* DNA, by the means of MiSeq Illumina sequencing platform, was 0.05 ng corresponding with a concentration of 10^4^ cfu/mL. Hence, the detection limit of the newly constructed primers was considered 10^4^ cfu/mL.

### 3.6. Resolution of Partial groEL Gene of Lactobacillus by Analyzing Samples from Varied Habitats

To evaluate the overall performance and resolution of the *Lactobacillus* new primers in different ecosystems, the *Lactobacillus* diversity of 24 samples, including six human fecal samples, six rat fecal samples, six mouse fecal samples, and six fermented yak milk samples was analyzed. Moreover, the V3–V4 region of 16S rRNA amplicons of these samples were sequenced. The 16S rRNA gene method assigned approximately 0.05–25% of the reads from the stool samples to the *Lactobacillus* genus, and the *Lactobacillus* species composition was not clear due to the limited resolution of the universal primer set 341F/806R. In contrast, using the *groEL* gene-based method, nearly all of the amplified sequences clustered as *Lactobacillus* and classified down to species level. Table 1 shows the microflora composition of four different habitats on the basis of 16S rRNA profiling method. Table 2 shows the species composition of the habitats on the basis of *groEL* gene profiling method. The values represent the average of the relative abundance of the microbes of six samples, and the value in parentheses is the standard error of the sample. The relative abundance values of genera and species less than 0.01 are omitted.

Further, using the *groEL* gene profiling, *L. mucosae* accounting for about 60% lactobacilli was found to be the most abundant *Lactobacillus* in people’s intestinal tract, followed by *L. gasseri* and *L. salivarius* (Table 2). In contrast, partial 16S rRNA gene profiling revealed that the *Lactobacillus* genus accounted for less than 3% of the human intestinal flora, the average is 1% (Table 1).

Analysis of genome DNA from six rat and six mouse fecal samples based on novel primer set Lac_*groEL*_F/Lac_*groEL*_R suggested the usefulness of this method for accurate cataloging of *Lactobacillus* species. In particular, *L. johnsonii* and *L. intestinalis* showed the highest relative abundance among the *Lactobacillus* population in the rat gut (Table 2), whereas *L. johnsonii* and *L. reuteri* were shown to be the main *Lactobacillus* in mouse gut (Table 2). Interestingly, 16S rRNA gene profiling method showed that the *Lactobacillus* genus accounted for more than 10% of the microbes in the rat and mouse intestines (Table 1).

In addition to using the method developed in this paper for studying the composition of lactobacilli in complex ecosystems, the formation of *Lactobacillus* species was also investigated in a relatively simple system. Tibetan people in Qinghai, China, prepare kurut using traditional methods. Six kurut samples were profiled using Lac_*groEL*_F/Lac_*groEL*_R, and the results showed that *L. delbrueckii* subsp. *bulgaricus* was the most abundant *Lactobacillus* species. Meanwhile, 16S rRNA gene profiling revealed that more than 82% of the microbes in kurut are *Lactobacillus*, followed by the Streptococcus genus at approximately 15% (Table 1).

Notably, OTU analysis of the results obtained from partial *groEL* and 16S rRNA gene profiling methods demonstrated 97% identity. *Lactobacillus* species with a relative abundance of less than 0.01 in each sample were excluded as “other *Lactobacillus* <1%” in the table, and OTU which could not be clustered as *Lactobacillus* was shown as “unassigned” when the results were based on *groEL* gene profiling method. The genera with a relative abundance of <1% in each sample were excluded as “*other Genera* <1%” in the table, and unassigned genera whose relative abundance were less than approximately 5% includes OTUs that were temporarily not recognized as any other genus when the results were based on 16S rRNA profiling method.

## 4. Discussion

To design a *Lactobacillus*-specific primer pair using MiSeq Illumina sequencing platform, several criteria need to be satisfied: first, the target gene must be present across all *Lactobacillus* species; second, the target gene should be sufficient to distinguish different species; third, the target gene should involve a highly variable region flanked by conserved regions on both sides; fourth, the region targeted for amplification within the target gene should be less than 500 bp long; and fifth, sufficient number of target gene sequences should be available [15,31]. The primer pair designed in this study met these criteria.

The detection limit of Lac_*groEL*_F/Lac_*groEL*_R was 10^4^ cfu/mL estimated from the means of MiSeq Illumina sequencing platform. Notably, in one previous study, the detection limit of *Lactobacillus* ITS profiling was 1.85 × 10^3^ cells/µL [10], but ITS marker has different operon copy number within a genome which may skew the diversity estimates of bacterial communities. Since *groEL* is a single-copy gene in *Lactobacillus* species, the estimates obtained in this study could be used as a reference. The assessment of detection specificity using the *Lactobacillus groEL* profiling method found that *Lactobacillus*, *Pediococcus*, *Weissella,* and *Leuconostoc* genus could be distinguished, but non-*Lactobacillus* Genus Complex could not be detected, indicating that the primers Lac_*groEL*_F/Lac_*groEL*_R are not specific only for *Lactobacillus*. It is not clear that *Lactobacillus* present in the microbial flora of the sample if the results merely based on the PCR amplification and gel imaging. Further confirmation would be needed by sequencing and alignment.

To evaluate the performance of *groEL* gene profiling method, *Lactobacillus* species diversity was assessed in different ecosystems. The results showed the composition and abundance of *Lactobacillus* species in people’s intestinal tract varied between different people. *L. mucosae* had the highest relative abundance of *Lactobacillus* in the intestine of Chinese, inconsistent with the previous finding that *L. acidophilus* and *L. helveticus* were the two dominant species in the gut of adult humans [32]. This discrepancy between the results could be explained by the finding that gut microbial composition differs between individuals based on internal and external factors such as diet, ethnicity, age, and physiological condition [33,34,35]. Furthermore, *L. delbrueckii* subsp. *bulgaricus* showed the highest relative abundance among *Lactobacillus* species in fermented yak milk samples, consistent with previous results [21]. The evaluation of the species diversity of *Lactobacillus* under different ecological environments is expected to provide valuable information for promoting the application of *Lactobacillus* for industrial production and therapeutic purpose.

There have been many classification methods since *Lactobacillus* was introduced in 1901, and it is likely that no single gene can distinguish between all *Lactobacillus* species. Thus, similar to most methods, *groEL* gene profiling is not without drawbacks. Cross-alignment analysis showed that lactobacilli have extremely high phylogenetic diversity (Table S5). Notably, the *groEL* sequences downloaded from the chaperonin sequence database (cpnDB) includes two strains of *Lactobacillus plantarum* and *Lactobacillus rumis* which are misclassified as *Lactobacillus salivarius*. In fact, *Lactobacillus plantarum* cannot be identical with *Lactobacillus salivarius* since these species are so distant on the basis of *groEL* gene sequence. Table 3 showed that 9 species shared *groEL* gene sequence identity over 97% with one or more *Lactobacillus* species. Thus, caution should be exercised when identifying these *Lactobacillus* species using the *groEL* gene as a genetic marker. The combination of *groEL* gene profiling with profiling based on 16S rDNA, *rpoB*, *clpC,* or any other gene may be more effective in distinguishing between *Lactobacillus* species. Moreover, complete genomic information, including that on *groEL* gene sequences, of some *Lactobacillus* species is not available. These species would be labeled as “unassigned *Lactobacillus*” when annotated in database. Thus, the database needs to be updated regularly when new species are discovered to enable the identification of all *Lactobacillus* species. Furthermore, *Pediococcus*, *Weissella*, and *Leuconostoc* genus produced corresponding fragments when using the degenerate primers for PCR amplification, indicating that the primers may be effective when assessing genera that are closely related to *Lactobacillus* genus.

## 5. Conclusions

In conclusion, a newly designed primer pair based on *groEL* gene could distinguish *Lactobacillus* species from other non-*Lactobacillus* Genus Complex bacterial species, with a detection limit of 10^4^ cfu/mL. The results of characterization of the *Lactobacillus* population in different ecosystems, such as human, rat, and mouse gut and traditional fermented yak milk, demonstrated the efficacy of this new *groEL* gene-based profiling method, indicating its applicability for academic research and industrial purposes.

## Figures and Tables

**Table 1:** 16S rRNA profiling method of the microflora composition of four different habitats ^1^.

	Samples	Human	Rat	Mice	Kurut
Genus					
*Enterobacteriaceae*		0.28(0.12)	0(0)	0(0)	0(0)
*Streptococcus*		0.11(0.09)	0(0)	0.02(0.02)	0.15(0.03)
*Bacteroides*		0.1(0.05)	0.03(0)	0(0)	0(0)
*Bifidobacterium*		0.09(0.03)	0(0)	0.07(0.05)	0(0)
other genera <1%		0.09(0.02)	0.07(0)	0.05(0.01)	0.01(0)
*Blautia*		0.06(0.03)	0.01(0)	0(0)	0(0)
*Ruminococcaceae*		0.05(0.02)	0.06(0)	0(0)	0(0)
*Clostridiales*		0.04(0.03)	0.16(0.01)	0.12(0.03)	0(0)
*Lachnospiraceae*		0.04(0.01)	0.03(0.01)	0.04(0.02)	0(0)
*Clostridiaceae*		0.03(0.01)	0.01(0)	0(0)	0(0)
*Collinsella*		0.02(0.01)	0(0)	0(0)	0(0)
*Enterococcus*		0.02(0.01)	0(0)	0.01(0)	0(0)
*Erysipelotrichaceae*		0.01(0)	0(0)	0(0)	0(0)
*Eubacterium*		0.01(0)	0(0)	0(0)	0(0)
*Lactobacillus*		0.01(0)	0.13(0.02)	0.14(0.02)	0.82(0.03)
*Mitsuokella*		0.01(0.01)	0(0)	0(0)	0(0)
*Parabacteroides*		0.01(0)	0(0)	0(0)	0(0)
*Prevotella*		0.01(0.01)	0.08(0.01)	0(0)	0(0)
*Ruminococcus*		0.01(0)	0.03(0)	0.05(0.01)	0(0)
*Adlercreutzia*		0(0)	0(0)	0.03(0.01)	0(0)
*Aerococcaceae*		0(0)	0(0)	0.01(0)	0(0)
*Aerococcus*		0(0)	0(0)	0.06(0.04)	0(0)
*Pseudomonas*		0(0)	0(0)	0(0)	0.01(0)
*Bacteroidales*		0(0)	0.17(0.01)	0.13(0.06)	0(0)
*Coprococcus*		0(0)	0.01(0)	0(0)	0(0)
*Desulfovibrionaceae*		0(0)	0.01(0)	0(0)	0(0)
*Helicobacteraceae*		0(0)	0.01(0)	0(0)	0(0)
*Planococcaceae*		0(0)	0(0)	0.03(0.01)	0(0)
*Pseudomonas*		0(0)	0(0)	0.01(0)	0(0)
*Oscillospira*		0(0)	0.04(0)	0.01(0)	0(0)
*Peptostreptococcaceae*		0(0)	0.05(0.01)	0(0)	0(0)
*Staphylococcus*		0(0)	0(0)	0.21(0.07)	0(0)
*Rikenellaceae*		0(0)	0.04(0.01)	0(0)	0(0)
*Turicibacter*		0(0)	0.02(0)	0.01(0)	0(0)
unassigned genera		0(0)	0.04(0.01)	0.01(0)	0(0)

^1^ The values represent the average of the relative abundance of the microbes of six samples, and the value in parentheses is the standard error of the sample. The relative abundance values of genera and species less than 0.01 are omitted.

**Table 2:** *groEL* gene profiling method of the species composition of four different habitats ^1^.

	Samples	Human	Rat	Mice	Kurut
Species					
*L. mucosae*		0.67(0.04)	0(0)	0.01(0.01)	0(0)
*L. gasseri*		0.06(0.02)	0(0)	0(0)	0(0)
*L. salivarius*		0.06(0.01)	0(0)	0(0)	0(0)
*other Lactobacillus* <1%		0.06(0.01)	0(0)	0(0)	0(0)
*L. oris*		0.05(0.03)	0.01(0)	0(0)	0(0)
*L. rhamnosus*		0.03(0.01)	0(0)	0(0)	0(0)
*L. amylovorus*		0.02(0.01)	0(0)	0(0)	0(0)
*L. casei*		0.01(0)	0(0)	0(0)	0(0)
*L. crispatus*		0.01(0)	0(0)	0(0)	0(0)
*L. plantarum*		0.01(0.01)	0.01(0)	0.01(0)	0(0)
*L. vaginalis*		0.01(0.01)	0(0)	0(0)	0(0)
*L. acidophilus*		0(0)	0(0)	0.1(0.03)	0(0)
*L. fermentum*		0(0)	0.05(0.05)	0(0)	0(0)
*L. reuteri*		0(0)	0.1(0.04)	0.11(0.02)	0(0)
*unassigned Lactobacillus*		0(0)	0.07(0.02)	0.1(0.02)	0(0)
*L. delbrueckii subsp. bulgaricus*		0(0)	0(0)	0(0)	0.97(0.03)
*L. helveticus*		0(0)	0(0)	0(0)	0.03(0.03)
*L. intestinalis*		0(0)	0.33(0.1)	0.01(0)	0(0)
*L. johnsonii*		0(0)	0.4(0.11)	0.65(0.04)	0(0)
*L. sp. L6*		0(0)	0.01(0)	0.01(0)	0(0)
*L. animalis*		0(0)	0.01(0)	0(0)	0(0)
unassigned species		0(0)	0.01(0)	0(0)	0(0)

^1^ The values represent the average of the relative abundance of the microbes of six samples, and the value in parentheses is the standard error of the sample. The relative abundance values of genera and species less than 0.01 are omitted.

**Table 3:** *Lactobacillus* species with *groEL* sequence identity of over 97% with another *Lactobacillus* species.

Species	*groEL* identity % with the Closet Species ^2^	Closest Species
*L. acidophilus*	100	*L. amylovorus*
*L. acidophilus*	99.8	*L. kitasatonis*
*L. amylolyticus*	99.6	*L. helveticus*
*L. buchneri*	99.8	*L. hilgardii*
*L. farciminis*	98.4	*L. formosensis*
*L. gasseri*	100	*L. paragasseri*
*L. johnsonii*	98.9	*L. taiwanensis*
*L. plantarum*	100	*L. pentosus*
*L. sakei*	99.3	*L. curvatus*
*L. zeae*	100	*L. paracasei*

^2^ The percentage shown in the table corresponds to the highest identity observed among *groEL* universal target sequences identified in strains of the two species compared.

## Supplementary Materials

The following are available online at https://www.mdpi.com/2073-4425/10/7/530/s1, Figure S1: A phylogenetic tree using Maximum Likelihood method on the basis of v3v4 region of 16S rRNA gene sequences for lactobacilli, Figure S2: Specificity of the newly designed primer pair in amplifying the selected partial *groEL* gene. Table S1: *Lactobacillus* strains used for primer specificity test, Table S2: 108 species of *Lactobacillus* used for primers design, Table S3: List of strains used for creation of the artificial sample, Table S4: List of *Lactobacillus* strains used in phylogenetic trees based on the v3v4 region of 16S rRNA and *groEL* gene, Table S5: MatGAT cross-alignment of *groEL* sequences of *Lactobacillus*.
