# Supplementary material for: groEL Gene-Based Phylogenetic Analysis of Lactobacillus Species by High-Throughput Sequencing
Source: Genes (Basel). 2019 Jul 12;10(7):530. doi: 10.3390/genes10070530 (PMC6678851; doi:10.3390/genes10070530)
Supplement: Supplementary file 1 [file genes-10-00530-s001.zip › supplementary/figure s1-s2_0628.pdf]

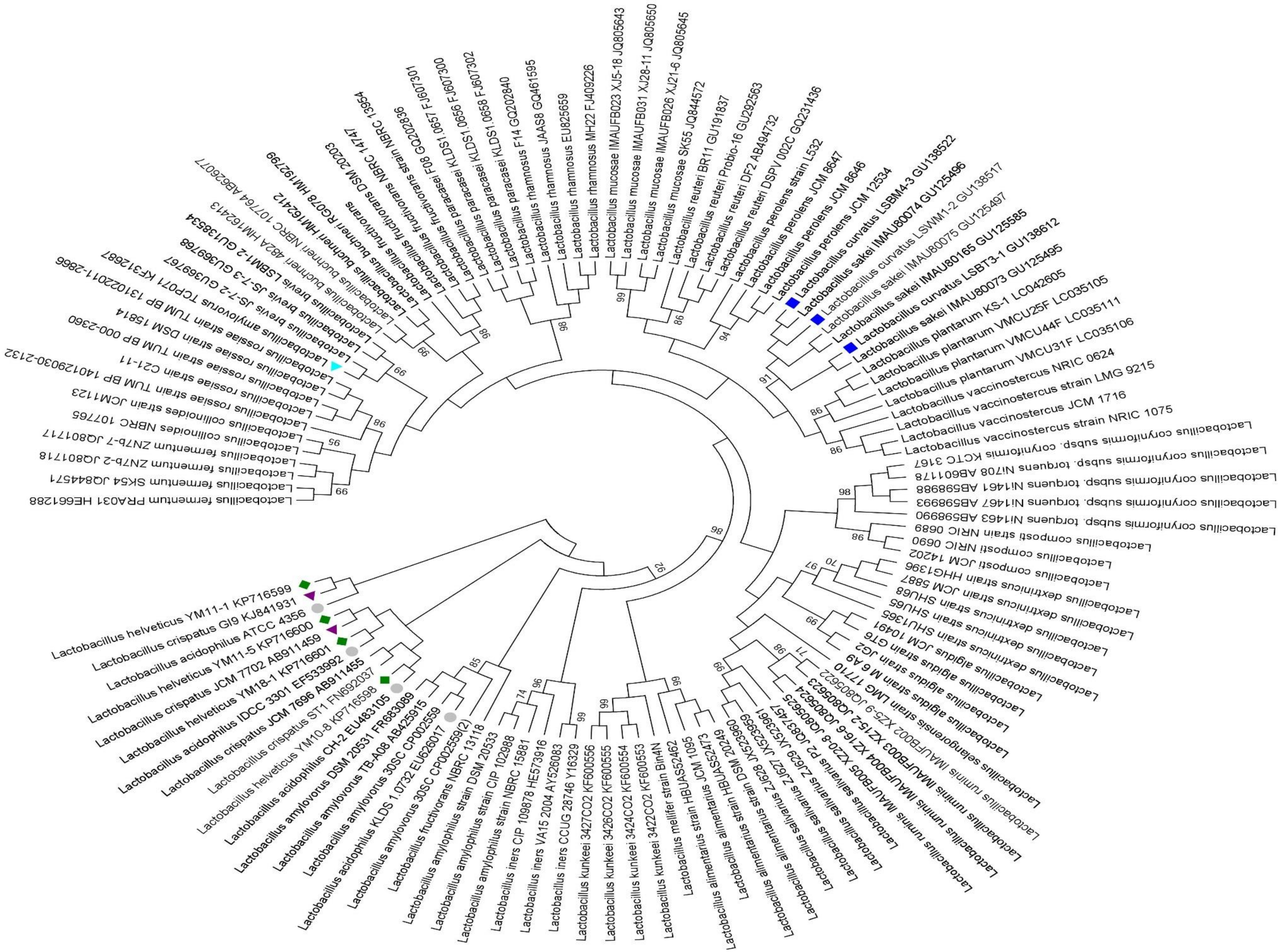

Figure S1: A phylogenetic tree using Maximum Likelihood method on the basis of v3v4 region of 16S rRNA gene sequences for lactobacilli,ML method was used with 1000 bootstrap, and bootstrap values over 70% were shown on the branches.

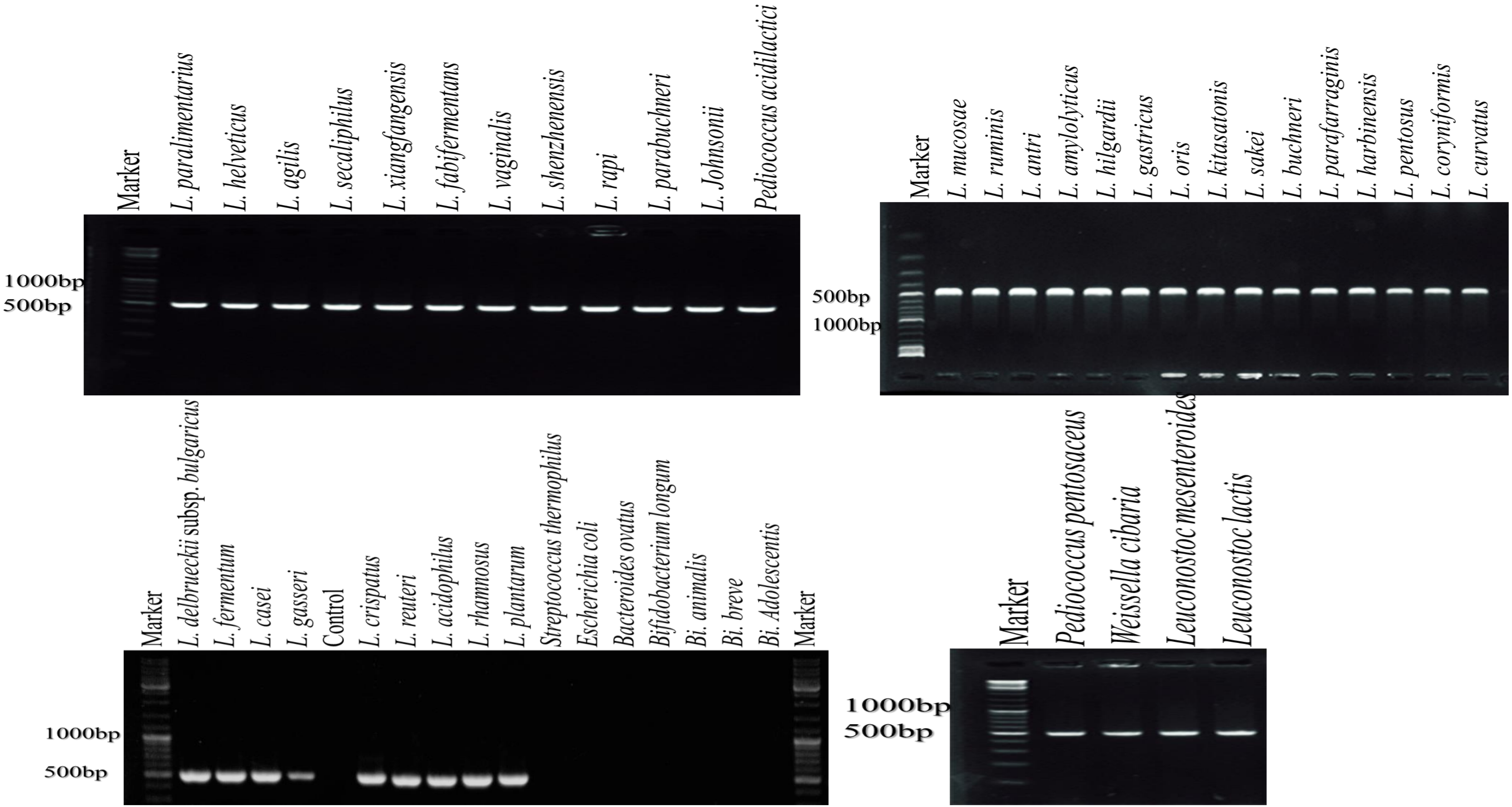

Figure S2: Specificity of the newly designed primer pair in amplifying the selected partial groEL gene.
